# Self-Sufficient Sensor Node Embedding 2D Visible Light Positioning through a Solar Cell Module

**DOI:** 10.3390/s22155869

**Published:** 2022-08-05

**Authors:** Irene Cappelli, Federico Carli, Ada Fort, Federico Micheletti, Valerio Vignoli, Mara Bruzzi

**Affiliations:** 1 Department of Information Engineering and Mathematical Sciences, University of Siena, 53100 Siena, Italy; 2 Department of Physics and Astronomy, University of Florence, 50019 Florence, Italy

**Keywords:** indoor positioning, visible light positioning, photovoltaic, energy harvesting, low power, ultra-low power, IoT, LoRaWAN

## Abstract

Nowadays, indoor positioning (IP) is a relevant aspect in several scenarios within the Internet of Things (IoT) framework, e.g., Industry 4.0, Smart City and Smart Factory, in order to track, amongst others, the position of vehicles, people or goods. This paper presents the realization and testing of a low power sensor node equipped with long range wide area network (LoRaWAN) connectivity and providing 2D Visible Light Positioning (VLP) features. Three modulated LED (light emitting diodes) sources, the same as the ones commonly employed in indoor environments, are used. The localization feature is attained from the received light intensities performing optical channel estimation and lateration directly on the target to be localized, equipped with a low-power microcontroller. Moreover, the node exploits a solar cell, both as optical receiver and energy harvester, provisioning energy from the artificial lights used for positioning, thus realizing an innovative solution for self-sufficient indoor localization. The tests performed in a ~1 m^2^ area reveal accurate positioning results with error lower than 5 cm and energy self-sufficiency even in case of radio transmissions every 10 min, which are compliant with quasi-real time monitoring tasks.

## 1. Introduction

Nowadays, accurate indoor positioning (IP) is one of the most ambitious challenges. While in the outdoor positioning, the global positioning system (GPS) is the dominant navigation technology, the same cannot be said for metropolitan and indoor applications, where its pervasive coverage and its high precision are strongly degraded by the presence of walls, furniture and obstacles. Indeed, one of the main aspects characterizing IP is the great heterogenicity of the indoor environment involved: the geometry, the type of obstacles and the required position accuracy greatly vary according to the application proposed and play an important role on the choice of the localization approach. This has led to the development of indoor positioning systems (IPSs) based on different techniques, extensively treated in the literature with surveys and reviews that compare their strengths and weaknesses [1,2,3,4,5,6,7,8].

Radio frequency technologies (e.g., radio frequency identification (RFID), global system for mobile communication (GSM), Wi-Fi, Bluetooth, Zigbee and long range wide area network (LoRaWAN)), sound-based technologies (e.g., ultrasounds and audible sounds), optical technologies (e.g., infrared and visible light) and magnetic field navigation are examples of commonly experimented indoor localization techniques relying on the presence of uniquely identified transmitters (e.g., Wi-Fi access points, light sources and Bluetooth beacons) as anchors to localize the mobile receiving target (i.e., an object with a receiver mounted on board). Some of these technologies as Wi-Fi or visible light are advantageous since they exploit existing infrastructures, thus requiring lower installation and maintenance costs with respect to positioning systems using specialized transmitters. Other approaches imply the usage of sensors mounted on the target and providing absolute localization independently from the presence of reference points; nevertheless, frequent recalibration and filtering procedures are required. Some examples are camera-based systems [9] and inertial navigation systems (INSs) [10,11], which incorporate accelerometers and gyroscopes to retrieve the linear acceleration and the rotational rate of the target.

Depending on the degree of precision desired, different localization strategies can be followed. The simplest approach for estimating the “raw” position is that of proximity detection, which gives information about proximity to a specific anchor instead of giving a precise position in the reference space. This approximate localization strategy finds application only in those contexts where a rough knowledge of the position is required (e.g., place recognition). If a higher level of accuracy is needed, triangulation and fingerprint methodologies can be adopted. The fingerprint method requires an offline training phase for collecting measurements or simulating data in several tests points, thus creating a database or a look up table of the operating space. Starting from the knowledge of the training set, other test points can be inferred, resorting to machine learning techniques or statistical methods. The major weaknesses of this approach are the time-consuming calibration phase, whose duration increases with the desired granularity, and the strong dependence on the specific test environment, requiring new time-consuming trainings every time it changes. The triangulation approach exploits the geometrical properties of the triangles formed by the target and the anchors to infer the target location. It comprises two different methods: angulation—which exploits the estimated angles between the target and multiple reference anchors—and lateration—which uses the estimated distances between the target and multiple reference anchors. The main quantities measured in these localization strategies are Angle of Arrival (AoA) for angulation, and Time of Arrival (ToA), Time Difference of Arrival (TDoA) and Received Signal Strength (RSS) for lateration. The first three techniques provide maximum position accuracy to the detriment of higher system cost and greater complexity. On the other side, the RSS-based method is site-specific and can be affected by errors in the estimation of the path loss model, due to signal reflections, shaded areas and obstacles between the receiver and the transmitters; moreover, some parameters of the model must be appropriately estimated during a calibration procedure to reduce errors in the subsequent localization phase.

Some possible application fields of IP are healthcare (e.g., monitoring of medical equipment and ambulating patients in hospitals), industry (e.g., asset tracking, robot navigation, automated storage and workers localization in hazardous environments) and Internet of Things (IoT) (e.g., tracking of goods, people and vehicles in warehouses, public buildings or civil infrastructures). The optimized management of energy resources and the attention towards sustainability are nowadays trend topics that are gathering growing interest. The increasing of IoT devices in paradigms such as Smart Home, Smart Factory and Smart City made more urgent the need to design systems independent from the connection to the electricity grid and to the periodic replacement of disposable batteries, or at least to extend their lifetime. Several sources can be exploited to perform energy harvesting for wireless sensor nodes. Wind [12], electromagnetism [13], ambient radio frequency [14], microbial fuel cells [15], thermoelectricity [16] and vibrations [17] are some examples; nevertheless, the primacy belongs to the solar energy source. In particular, most of the applications exploit photovoltaic (PV) cells exposed to direct sunlight to provision energy for self-sufficient wireless sensor nodes [18]. However, there is a growing interest in the possibility of exploiting PV modules for energy harvesting also from diffused sunlight and from artificial light in indoor environments, both with white and colored spectrum [19,20,21,22].

For this reason, among the aforementioned IP technologies, visible light positioning (VLP) has arisen as valid solution for the deployment of low power localizable targets [1,2,7], simultaneously providing energy harvesting features thanks to the exploitation of the same light signals used for localization. Moreover, VLP-based IPSs offer good positioning accuracy with reduced cost and complexity both for the transmitting device—the existing lighting infrastructures can be exploited—and the receiving device—a commercial optical receiver with simple conditioning electronics can be used, especially in case of RSS approach.

In this paper, we propose a low power VLP system equipped with LoRaWAN connectivity and employing a small-sized PV module both for 2D indoor localization and energy provisioning. The designed prototype is conceived in a low power perspective in such a way to be compliant with the energy harvesting aspect. Indeed, resorting to a solar cell-based IPS is inherently a low power approach with respect to radio frequency positioning methods using power-consuming off-the-shelf receivers (e.g., Sub-GHz band or WiFi). Moreover, the electronic components are selected among families specifically thought for applications envisaging low consumption. The localization methodology adopted is lateration, performed by using the RSSs of three LED (light emitting diode) placed in known positions of the measurement area. The RSSs extraction is performed by means of fast Fourier transform (FFT) of the received light signal in order to recover the contribution of each lamp, univocally identified by a unique driving frequency. The coordinates extraction is directly performed on board of the target itself and sent via LoRaWAN, making it available on a server, thus avoiding post-processing operations at the backend side as is the case of RSS-based solutions using radio technologies. Only one measurement point in the center of the measurement area subtended by the three LEDs is needed to calibrate the localization algorithm.

Considering that the system is designed keeping in mind the low-cost and low-power requirements, it will not intrinsically reach the sub-cm accuracy of expensive and highly precise devices. However, it must be accounted that in a real context indoors, the achievement of this level of accuracy with localization is quite unrealistic; other factors, such as the presence of obstacles, shadows and reflections inevitably lead to a degradation of the performance or even to the total impossibility of localization.

This manuscript is organized as follows: in Section 2, the state of the art of VLP is surveyed, focusing, in particular, on energy autonomous solutions using solar cells, and the originality of the proposed system is highlighted; Section 3 gives a description of the adopted localization strategy; Section 4 describes the materials and methods used for the measurements, while Section 5 illustrates the system architecture; in Section 6, the experimental outcomes of the tests are presented and discussed; and, finally, in Section 7, some conclusive remarks are provided.

## 2. State of the Art

IPSs based on VLP using LEDs have been widely studied in the literature [1,2,7]. The main distinction can be made on the basis of the used receiver; that can be a camera in vision-based applications or an optical sensor as a photodiode or a PV module. In particular, we will focus on those works based on solar cells, highlighting our contribution to the state of the art and the novelty of the proposed system.

Some applications involving solar cells as photodetector concern visible light communication (VLC) [23,24,25] or more in general optical wireless communication (OWC) [26] rather than VLP; therefore, with respect to the proposed system, no localization task is performed. In these works, the light sources are used for a dual purpose of illumination and high frequency data transmission, replacing the radio communication medium with the optical one, which can be employed in those critical contexts, as hospitals, where radio frequency signals could interfere with machinery and instrumentation. The compatibility of the communication with the energy harvesting feature is also investigated, exploiting the DC component of the received optical signal that is normally discarded for communications tasks. Even though the photodiode is usually preferred to the solar cell as photodetector, several works exist in the literature treating VLP with solar cells [27,28,29,30,31], although most of the time the harvesting aspect is not accounted at all or, at most, is mentioned as a possibility without presenting a complete integrated system. The application in [27] exploits an RSS-based trilateration positioning method to recover the mutual distances between transmitting LEDs and receiver with a mean error of about 10 cm, which is the same approach presented in our work. However, the signal demodulation and the positioning algorithm are implemented offline on a PC without presenting an integrated system performing autonomous indoor localization. Moreover, the energy harvesting aspect is not deeply analyzed, but just presented as a possible future development. Even in [29], the authors do not investigate the harvesting part, but just simulate the fact that the proposed system is capable of simultaneous communication and energy gathering. Moreover, the LEDs transmit their identification numbers by on–off keying (OOK) instead of using a unique frequency allocation methodology as we propose, implying a much more complex customized lighting hardware since additional equipment as microprogram control unit (MCU), synchronization and code modulation circuits must be integrated. Different methodologies are presented in [30,31] where machine learning models are trained using several fingerprints to attain localization. In particular, in [30], the authors present a wearable prototype integrating three energy harvesters (i.e., solar cell, piezo and thermoelectric generators) and performing place recognition. Therefore, with respect to the proposed system, no exact coordinate estimation is achieved, in fact the prototype acts as a classifier recognizing places on the basis of the electricity generated and the user movement. In [31], the human localization is attained from the radiation changes measured by fixed solar cells and dependent on the human position in the room. The main disadvantage of machine learning approaches is that several fingerprints must be previously collected to train the algorithm, whilst our proposed method needs just one calibration measurement in the center of the measurement area subtended by each triad of LEDs. Finally, in [10], the authors propose a more complex solution composed of an INS combining an IMU (inertial measurement unit) with a solar cell used both for energy harvesting and for data fusion to correct IMU’s drift errors.

Therefore, to our knowledge, our contribution is the first one presenting an integrated energy self-sufficient LoRaWAN-based device performing on board 2D indoor positioning using a PV module both for localization and for artificial light energy harvesting. Moreover, the great advantage of this application relies on its low complexity in the demodulating side, which can be entirely realized resorting to off-the-shelf components making the proposed system a valid solution for large scale deployments.

## 3. Localization Algorithm

The presented approach exploits three LEDs, univocally identified by a unique operating frequency, as anchors to recover the 2D position of the optical receiver from the knowledge of the LEDs coordinates in the measurement space and from the extracted RSSs. In particular, a PV module is used as optical receiver, producing a photocurrent converted into a voltage directly proportional to the incident optical power of each LED (i.e., the RSSs). The fact of having assigned a unique frequency to each LED made it possible to avoid, during the reception phase, interference between the light signals coming from the three sources at the same time. This approach also allows to ignore in the demodulation phase the contribution of the background light at a frequency different from those transmitted or which acts as an average value on the detected signal.

**Figure 1:**
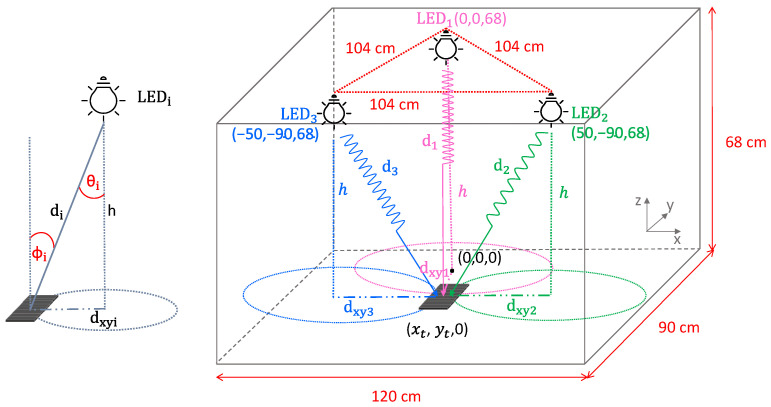
Geometrical representation of the adopted localization approach.

The geometrical representation of the set-up together with the reference system is reported in Figure 1: the three LEDs are placed on the same plane while the optical receiver lays on the floor at a fixed distance h from the LEDs; the origin of the 3D measurement space is located in the projection point of ${\mathrm{LED}}_{1}$ on the floor.

The localization task is attained in three distinct phases:

1. RSSs extraction through FFT algorithm;
2. Lambertian optical channel model inversion;
3. Lateration method to predict the coordinates of the target ($x_{R},y_{R},z_{R}$).

The output of the FFT algorithm is a vector containing the amplitudes of the received signal in correspondence of the unique frequencies identifying the three light sources, $V_{\mathrm{Ri}}$. Starting from these intensities, the distances $d_{i}$ with i = 1,2,3 between optical receiver and ${\mathrm{LED}}_{i}$ are derived inverting Equation (1), which describes the Lambertian optical channel for each LED [32,33].

(1) $$P_{\mathrm{Ri}}=P_{\mathrm{Ti}}\frac{\left(m_{T}+1\right)}{2{\pi d}_{i}{}^{2}}\mathrm{AT}\left(\phi_{i}\right)G\left(\phi_{i}\right)\cos^{m_{T}}\left(\theta_{i}\right)\cos^{m_{R}}\left(\phi_{i}\right)\;$$

$P_{\mathrm{Ri}}$ are the measured RSSs—in our application directly derived from the measured voltages $V_{\mathrm{Ri}}$ extracted with the FFT algorithm; $P_{\mathrm{Ti}}$ are the transmitted optical powers of each LED; A is the optical receiver active area; $T\left(\phi_{i}\right)$ and $G\left(\phi_{i}\right)$ are the optical filter and optical concentrator gains in case they are present; $m_{T}$ and $m_{R}$ are the Lambertian mode numbers of LED and optical receiver derived as mR=ln2lncosϕ12=1.12 and mT=ln2lncosθ12=0.74 where ϕ12 and θ12 are the half sensitivity receiver and transmitter angles; and $\cos\phi_{i}$ and $\cos\theta_{i}$ are the cosines of the transmitted angle between light and LED axes and of the receiving angle between light and optical receiver axes. A simplification of the model is done assuming to know h and that the LEDs and optical receiver axes are always parallel, in this way $\theta_{i}$ and $\phi_{i}$ have the same value equal to $\cos^{-1}\frac{h}{d_{i}}$, thus obtaining $d_{i}$ as

(2) $$\begin{array}{c} d_{i}=\sqrt[{m_{R}+m_{T}+2}]{P_{\mathrm{Ti}}\frac{\left(m_{T}+1\right)}{2{\pi P}_{\mathrm{Ri}}}\mathrm{AT}\left(\phi_{i}\right)G\left(\phi_{i}\right)h^{\left(m_{R}+m_{T}\right)}}\;\;\;\\ =\sqrt[{m_{R}+m_{T}+2}]{K_{i}\frac{\left(m_{T}+1\right)}{2{\pi V}_{\mathrm{Ri}}}Ah^{\left(m_{R}+m_{T}\right)}} \end{array}$$

Some parameters of this model are obtained from the knowledge of the physical characteristics of LEDs and solar cell (i.e., A, $m_{R}$, $m_{T}$), others contribute to a calibration parameter $K_{i}$ estimated through a preliminary measurement performed in only one point of the grid and with the three LEDs on. The solar cell is placed in the centroid of the triangle identified by the projections of the LEDs on the floor, then by inverting (2), the $K_{i}$ i = 1,2,3 are derived, considering that the distances $d_{i}$ of the PV module from each LED are known a priori.

Once the distances are retrieved from the inversion of the mathematical model for light transmission and known the vertical height of the LEDs h, the horizontal distances $d_{{\mathrm{xy}}_{i}}$ on the receiver plane are extracted and then used to estimate the receiver coordinate ($x_{R},y_{R},z_{R}$) with respect to the fixed reference system. This is performed through the lateration method, which estimates the 2D position as intersection on the plane of the three circumferences centered in the projection point of the LEDs on the plane and with radius equal to the estimated horizontal distances $d_{{\mathrm{xy}}_{i}}$ (see Figure 1 for a geometrical representation). The system of the three quadratic equations is

(3) $$\left\{\begin{array}{c} {\left(x_{R}-x_{1}\right)}^{2}+{\left(y_{R}-y_{1}\right)}^{2}+{\left(z_{R}-z_{1}\right)}^{2}=d_{{\mathrm{xy}}_{1}}^{2}\;\\ {\left(x_{R}-x_{2}\right)}^{2}+{\left(y_{R}-y_{2}\right)}^{2}+{\left(z_{R}-z_{2}\right)}^{2}=d_{{\mathrm{xy}}_{2}}^{2}\\ {\left(x_{R}-x_{3}\right)}^{2}+{\left(y_{R}-y_{3}\right)}^{2}+{\left(z_{R}-z_{3}\right)}^{2}=d_{{\mathrm{xy}}_{3}}^{2} \end{array}\right.$$

where ($x_{i},y_{i},z_{i}$) are the known positions of the LEDs in the measurement space. In our application, $z_{1}=z_{2}=z_{3}=h$ and $z_{R}=0$. With few mathematical manipulations, the previous system can be written in the following form, becoming a solvable linear system of two equations and ($x_{R},y_{R}$) unknowns.

(4) $$Cx=B\;\mathrm{with}\;C=\left[\begin{array}{cc} \left(x_{2}-x_{1}\right) & \left(y_{2}-y_{1}\right)\\ \left(x_{3}-x_{1}\right) & \left(y_{3}-y_{1}\right) \end{array}\right],\;x=\left[\begin{array}{c} x_{R}\\ y_{R} \end{array}\right]\;\mathrm{and}\;B=\left[\begin{array}{c} \left(d_{{\mathrm{xy}}_{1}}^{2}-d_{{\mathrm{xy}}_{2}}^{2}+x_{2}^{2}+y_{2}^{2}-x_{1}^{2}-y_{1}^{2}\right)/2\\ \left(d_{{\mathrm{xy}}_{1}}^{2}-d_{{\mathrm{xy}}_{3}}^{2}+x_{3}^{2}+y_{3}^{2}-x_{1}^{2}-y_{1}^{2}\right)/2 \end{array}\right]$$

The tests presented later in the paper are performed in the rectangular area enclosing the triangular projection of the LEDs on the floor; however, the proposed localization approach can be generalized to a realistic wider deployment following the idea of dividing the space of interest into triangular cells each identified by three LEDs chopped at unique frequency. In situations where more LEDs should be deployed, it may happen that in a cell, in addition to the three LEDs identifying it, even the LEDs of nearby cells will be visible. To handle this situation, the proposed methodology considers only the three highest FFT-peaks among those detected hence, supposing no obstacles in between, these peaks will coincide for sure with the LEDs identifying the cell. Moreover, one calibration point must be taken in the centroid of each cell, hence the complexity of the calibration procedure in the overall space will depend on the number of cells. Resorting to a wider measurement area has no effect on the computational operations performed by the microcontroller, except that increasing the number of cells will increase the size of the look up tables stored in the MCU containing the associations between LEDs and coordinates, and $K_{i}$ and cells.

## 4. Materials and Methods

In this Section, the instruments and the procedures used to perform the localization and self-sufficiency tests are presented.

### 4.1. Light Sources

The optical sources employed to perform the localization task are three 4000 K LEDs, produced by Cree Lighting, with 115° viewing angle, CRI 80, 1563 lm and spectral power distribution in the visible range. They are driven by a properly designed and realized LED driver dealing with the power supply and the control of the driving signals coming from three Agilent 33220A arbitrary waveform generators. The luminous signals issued by the LEDs are chopped at different frequencies (i.e., $f_{1}$ = 1100 Hz, $f_{2}$ = 825 Hz, $f_{3}$ = 975 Hz). These frequencies, together with analog-to-digital converter (ADC) sampling frequency and number of samples used for the FFT computation, are conveniently chosen to respect several requirements: avoiding light flickering phenomena (signal frequency higher than 160 Hz), satisfying the PV module bandwidth (~3.5 kHz), preventing spectral leakage in the demodulation phase and minimizing the interference between the harmonic components of the resulting signal obtained as combination of the three luminous waves (more details about this last two points are reported in Section 5.2)

### 4.2. Measurement Set-Up

The three LEDs are positioned at 68 cm from the floor and perpendicularly to it, arranged at the vertices of an equilateral triangle with side equal to 104 cm, covering a localization area with dimensions 90 cm × 120 cm, representing a 1:5 scaled version of a real deployment (as depicted in Figure 1). This area is subdivided into 108 10 cm × 10 cm squares identifying 130 distinct equally spaced measurement points, which represent the same amount of possible 2D positions in the plane. The measurements are taken without a complete shielding of the testing area, since the proposed localization approach should be not affected by other indoor light sources. Considering all the three lights on (when in the absence of other external light sources), the average light intensity under each LED, measured using a RS-LM-1337 lx/fc photodiode-based light meter, is equal to about 500 lx, reaching a minimum value of 410 lx in the center of the grid, compatibly with the lighting specifications for indoor working environments.

**Figure 2:**
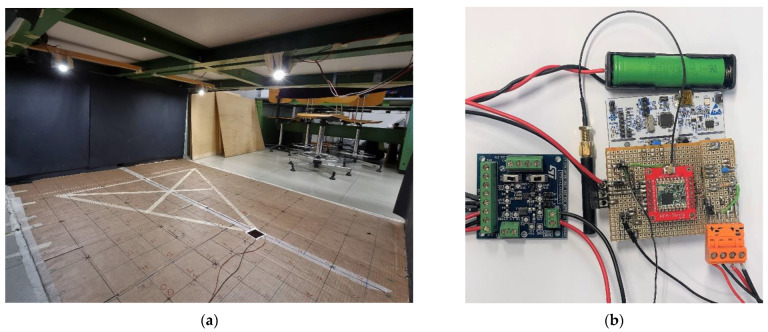
(**a**) The measurement area with the three LEDs and the localization grid at 10 cm, the ${\mathrm{LED}}_{1}$ is the one furthest to the right in correspondence of the PV module. (**b**) The proposed IPS in charge of demodulating the signal, extracting the 2D position and transmitting it via LoRaWAN protocol; the battery management board is on the left.

The measurement area with the localization grid at 10 cm, and the proposed IPS are shown in Figure 2 (a detailed description of the system architecture is provided in Section 5). The ${\mathrm{LED}}_{1}$ is the one furthest to the right in Figure 2a, in correspondence with the PV module used for localization.

### 4.3. 2D Indoor Positioning Tests

Several measurement campaigns are performed with the previously described measurement set-up in order to validate the localization algorithm implemented. During these tests the receiver is moved along the grid in steps of 30 cm and 10 cm covering the entire localization area or a subset of it; during its shift, the optical receiver is maintained parallel to the floor without admitting inclination with respect to the LEDs plane.

The 2D positions are estimated using the localization algorithm described in Section 3 and implemented on an on-board MCU. The estimated coordinates are then sent via LoRa and made available on a Server (i.e., a comprehensive description of the node architecture is reported in Section 5). The performance of the proposed VLP algorithm is finally evaluated with MATLAB 2021 (MathWorks, Natick, MA, U.S.).

### 4.4. Node Self-Sufficiency Tests

The energy self-sufficiency of the node is verified by monitoring the voltage level, $V_{\mathrm{Li}-\mathrm{Ion}},$ of the rechargeable Li-Ion battery mounted in the system and charged by the energy collected by the PV panel under the alternating luminous signals. The voltage is acquired by a Keysight 34470A multimeter (7 ½ digit resolution) controlled via LabVIEW with sampling period of 0.5 s.

## 5. System Architecture

**Figure 3:**
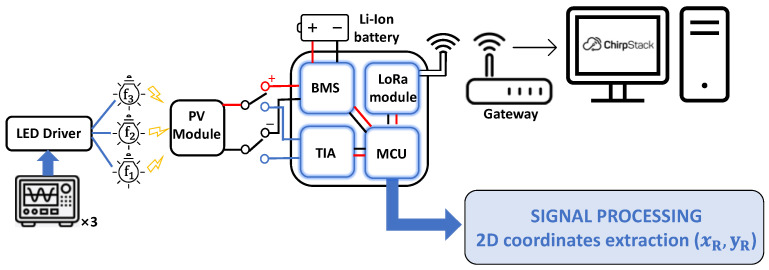
Architecture of the proposed positioning system: the transmitter (i.e., LED driver driven by three waveform generators, three LEDs), the receiver (i.e., the PV module with its conditioning circuit, the battery management system and the demodulating system—MCU and LoRa transceiver), the backend side (i.e., the LoRa gateway forwarding the transmitted packets to the server).

**Figure 4:**
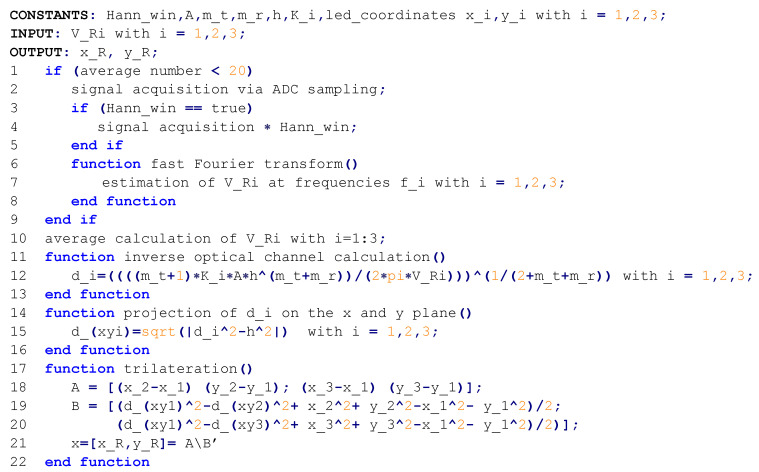
Pseudocode resuming the sequence of operations performed by the MCU to achieve the localization.

The architecture of the proposed system is depicted in Figure 3, while the pseudocode of the tasks performed by the MCU to attain the 2D positioning estimation is resumed in Figure 4.

At the receiver side, three main parts can be found: the optical receiver, comprising the solar cell and the conditioning circuit; the demodulating system, embedding the MCU and the LoRa transceiver to transmit the extracted 2D position; and the battery management system (BMS), dealing with the energy harvesting from the PV module used as light sensor. Low-power components and programming strategies have been selected in order to realize an energy self-sufficient localization device.

### 5.1. Optical Receiver

**Figure 5:**
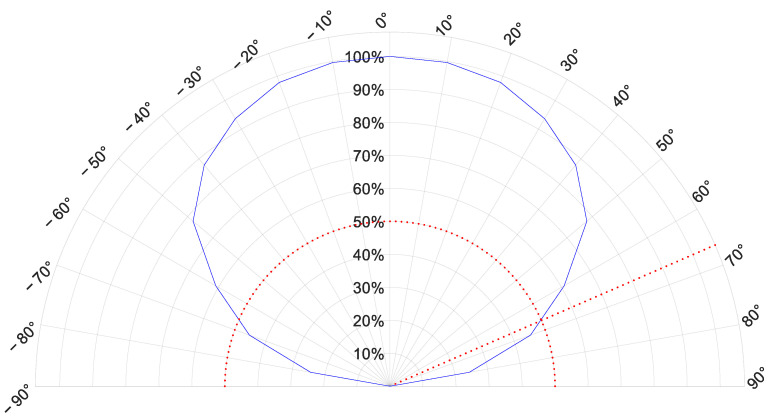
Relative sensitivity [%] versus the angular displacement of the PV module. The semicircle and the segment in red identify the angle of half sensitivity.

The mounted optical receiver is an amorphous silicon PV module manufactured by Panasonic and specifically thought for working under artificial light illumination. Indeed, the external quantum efficiency (EQE)’s response band of amorphous silicon is limited to the visible range with a maximum around 540 nm, well-matching the spectral distribution of the LEDs used as light sources. The PV module is made on a glass substrate and is composed of a series of 8 cells with an overall active area A = 23.6 ${\mathrm{cm}}^{2}$, it has nominal open circuit voltage ${\;V}_{\mathrm{OC}}$ = 4.9 V and short circuit current ${\;I}_{\mathrm{SC}}$ = 47.0 µA at 25 °C under 200 lx fluorescent light. Its angle of half sensitivity has been experimentally inferred tilting the module under the light source in steps of 10° and a value of ±67° has been found. The trend of the relative sensitivity with respect to the angular displacement is shown in Figure 5: the red-dotted semicircle and segment graphically identify the angle of half sensitivity. Moreover, the sensitivity trend suggests that the proposed methodology can handle inclinations of the PV module axis with respect to the floor in the order of ±10°.

**Figure 6:**
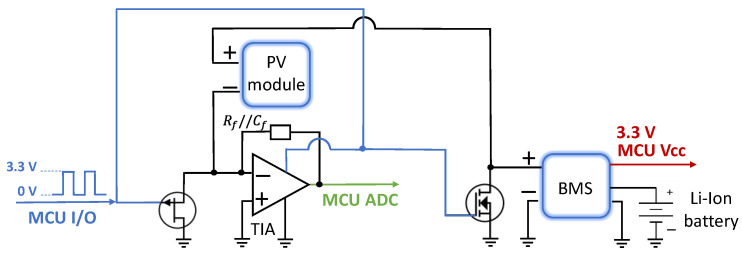
Implemented strategy, relied on a control signal from an I/O port of the MCU, to manage the same PV module both for energy harvesting and for localization estimation.

During the localization phase, by properly driving the two electronic switches mounted as shown in Figure 6, the cell is disconnected from the BMS and connected in a zero-bias configuration to the low-power operational amplifier TLV237 by Texas Instrument, mounted as transimpedance amplifier (TIA). The TIA presents a very low input impedance; therefore, it is able to measure the short circuit current provided by the PV module, and to convert it into a voltage dependent on the level of illumination incident on the device. The feedback impedance used is a parallel connection of a resistance $R_{f}=$ 15 kΩ and a capacitance $C_{f}=$ 15 pF.

### 5.2. Demodulating System

The output of the TIA is acquired via the ADC of a STM32L476RGT6 ARM Cortex M4 microcontroller mounted on a NUCLEOL476RG development board from ST Microelectronics. The microcontroller is in charge of digital processing the sampled voltages by performing the discrete Fourier transform (DFT) of the signal by means of FFT algorithm in order to extract the RSS of each light source. Once the three magnitudes are computed, the trilateration algorithm is performed outputting the estimation of the 2D coordinates of the optical sensor.

These data are then sent via LoRaWAN protocol to a LP68 Dragino gateway, which redirects the packets to a ChirpStack Server connected to a InfluxDB database. The LoRa low power radio module employed is a RFM95x equipped with a λ/8 antenna. The used radio settings are transmitting frequency 868 MHz, bandwidth (BW) 125 kHz, output power 5 dBm, coding rate (CR) 4/5 and spreading factor (SF) 7. Both CR 4/5 and SF 7 are chosen since they guarantee the lowest power consumption and the shortest time on air (working currents with peaks up to 45 mA for ~62 ms in transmission) to detriment of worse error correction at the reception and link margin reduction which, however, are not critical aspects in the proposed scenario where kilometric radio coverage is not required.

During the node self-sufficiency test, the MCU is programmed according to a sleep routine, which periodically activates the MCU for the time required to retrieve the 2D position of the object and send the data. Since the position is computed on the average of 20 implementations of the FFT algorithm, a run period of approximately 2.5 s can be considered. One digital I/O port is used both to turn on a MOSFET driving the LoRa module and to supply the TIA, in order to switch them off during the sleep periods of the MCU thus avoiding extra power consumption. In this way, the current absorption of the node in standby mode falls below the μA.

#### FFT Implementation

The number of points used for the FFT computation, and the ADC sampling frequency are conveniently chosen in accordance with the LEDs signals in order to theoretically avoid spectral leakage and frequency interference.

The number of samples required by the FFT algorithm is set to N = 4096 while the ADC sampling frequency is set to $f_{s}$ = 102,400 Hz, giving a frequency resolution Δf = 25 Hz and an overall observation window equal to 40 ms. This sampling frequency is conveniently chosen in such a way to have an integer number of periods of the three incoming signals in the observation window. Indeed, choosing the aforementioned values for N, $f_{s}$, $f_{1}$, $f_{2}$ and $f_{3}$ guarantees the minimization of the spectral leakage. However, a small error can still be observed if there is a misalignment among the clock of the microcontroller and the frequencies of the signals driving the LEDs. In this respect, some tests have been performed giving as an input of the MCU ADC square signals with known and fixed amplitude and frequencies in the range from 525 Hz to 50 kHz with steps of 1 Hz using the waveform generators used in the test bench; it was found that a misalignment of few mHz is present among the clocks, resulting in errors in the reconstruction of the signal amplitude. These small frequency errors, due to the quality of the quartz clocks in the test bench, have small effects on the FFT peak amplitude estimation accuracy. Nevertheless, in low-cost system a worse clock accuracy is expected, which can affect the FFT peak measurement accuracy. In fact, in on field deployments the lighting system will be driven by low-cost relaxation oscillators instead of signal generators. Note that with the chosen setting, a stable misalignment of 12.5 Hz results in a relative peak amplitude error of ~36%. If the clock remains stable, this issue can be compensated by the proposed IP algorithm with the computation of $K_{i}$ coefficients during the calibration procedure. On the other hand, errors that cannot be compensated by any calibration procedure are those caused by the interference between the spectra of the three LEDs signals, which, in principle, are eliminated in our system with the selected values of the three frequencies ensuring the acquisition of an integer number of periods for the three signals. Nevertheless, the clock inaccuracies introduce fractional parts of the periods to the acquired windows and cause interferences. This issue was counteracted by choosing $f_{1}$, $f_{2}$ and $f_{3}$ sufficiently distant in frequency (i.e., minimum 3 side lobes between two consecutive main lobes) and a Hann windowing operation has been added before FFT, reducing the discontinuity between subsequent windows, and thus mitigating the spectral leakage even in case of non-correct frequency generation.

In light of these considerations, a possible estimation of the maximum number of LEDs in one single room is 10; in the same room, it is not possible to have two or more LEDs with the same identifying frequency, that instead can be reused in a different room.

### 5.3. Battery Management Unit

The ultralow-power energy harvester and battery charger employed to collect energy from the three LEDs is the SPV1050 manufactured by ST Microelectronics, mounted on the STEVAL-ISV020V1 board. It is a commercial integrated circuit (IC) featuring boost charging from sources delivering at least 150 mV in order to charge a storage element, in the case study, a rechargeable 3.7 V 3400 mAh Li-Ion battery employed as power reserve. Undervoltage and overvoltage thresholds can be set through resistive dividers to protect the battery from overcharging and excessive drainage from the load. Indeed, the integrated buck converter provides a regulated voltage supply of 3.3 V used to power the MCU, which in turn drives the system electronics. Moreover, the IC offers an adjustable internal maximum power point tracking (MPPT) functionality, which samples the open circuit voltage of the energy source, $V_{\mathrm{OC}}$, for 400 ms every 16 s and set the working voltage to a preset percentage of $V_{\mathrm{OC}}.$ In the presented application, this ratio is set to 0.8$V_{\mathrm{OC}}$ which theoretically corresponds to the maximum power transfer condition for PV modules.

In the tested conditions of minimum illuminance (i.e., 410 lx), the average voltage and current at the operating point 0.8$V_{\mathrm{OC}}$ are $V_{\mathrm{OP}}$ = 4.38 V and $I_{\mathrm{OP}}$ = 70 µA, giving an average extracted power $P_{\mathrm{OP}}$ ≃ 305 µW.

Solid-state switches (MOSFET and JFET) are used to manage the same PV module both for energy harvesting and for localization estimation, thus preventing the reading of the light signal from being affected by the MPPT process of the BMS, which fixes a constant voltage at the terminals of the solar cell using a variable load and a switching strategy. As soon as the MCU wakes from standby mode, it enables the same I/O port used to pilot the LoRa module to drive the switches, which temporarily detach the cell from the BMS for few seconds and connect it to the TIA input; therefore, during this period, no energy is harvested. Once the position coordinates are sent via LoRaWAN and before the MCU re-enters standby mode, the I/O port is disabled, and the PV module is again used for energy provisioning. Moreover, this strategy assures that in case of deactivation of the load by the BMS (i.e., battery voltage under the undervoltage threshold) the PV module remains connected to the BMS input to guarantee battery recharging. The implemented configuration is depicted in Figure 6.

## 6. Experimental Results and Discussion

In this section, the results of the measurement campaigns are reported, performed using the measurement set-up and the methods described in Section 4. Two different tests can be identified: 2D indoor positioning tests—to assess the performance of the proposed localization algorithm—and node self-sufficiency tests—to evaluate the energy-autonomy capability of the node.

### 6.1. 2D Indoor Positioning Tests

**Figure 7:**
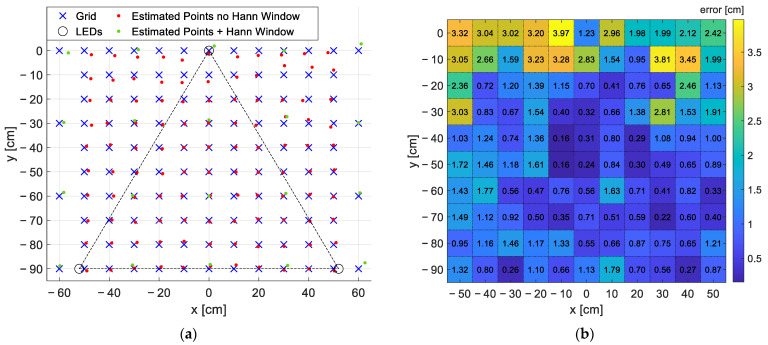
Results of the positioning test in 110 measurement points equally spaced by 10 cm. (**a**) Qualitative grid representation with the origin of the reference system fixed in correspondence of ${\mathrm{LED}}_{1}$: in blue the ‘true positions’ of the grid points, in red the coordinates estimated without windowing operation, in green the points estimated at steps of 30 cm using the Hann window, in black the circles identifying the LEDs positions (top of the grid ${\mathrm{LED}}_{1}$, bottom right ${\mathrm{LED}}_{2}$ and bottom left ${\mathrm{LED}}_{3}$) and the triangular area subtended by the LEDs. (**b**) Numeric representation of the error in each point as a heatmap, the values in the map report the exact error in cm.

To test the performance of the proposed IP technique, the receiver was moved over a grid, with steps of 10 cm covering a portion of 100 cm × 90 cm of the localization area (i.e., the vertices of 90 10 cm × 10 cm squares), by keeping the light sensor laid on the floor, i.e., with the optical axis parallel to the LEDs’ ones, and collecting a triad of LEDs RSSs in each measurement location. This information is then used by the on-board microcontroller to recover the 2D position. In Figure 7, two representations of the results of the positioning test are reported. Figure 7a shows, in red, the coordinates estimated without windowing operation together with the corresponding ‘true positions’ of the grid points in blue (the ‘true positions’ and the height *h* were measured by means of an independent technique using a measurement tape with an uncertainty of 0.5 cm). In the same figure, the points taken with steps of 30 cm, but using the Hann window, are represented in green, whereas the black circles identify the projections of the LEDs on the floor (top of the grid ${\mathrm{LED}}_{1}$, bottom right ${\mathrm{LED}}_{2}$ and bottom left ${\mathrm{LED}}_{3}$). Finally, as in Figure 1, the origin of the reference system is fixed in correspondence of ${\mathrm{LED}}_{1}$ projection.

**Figure 8:**
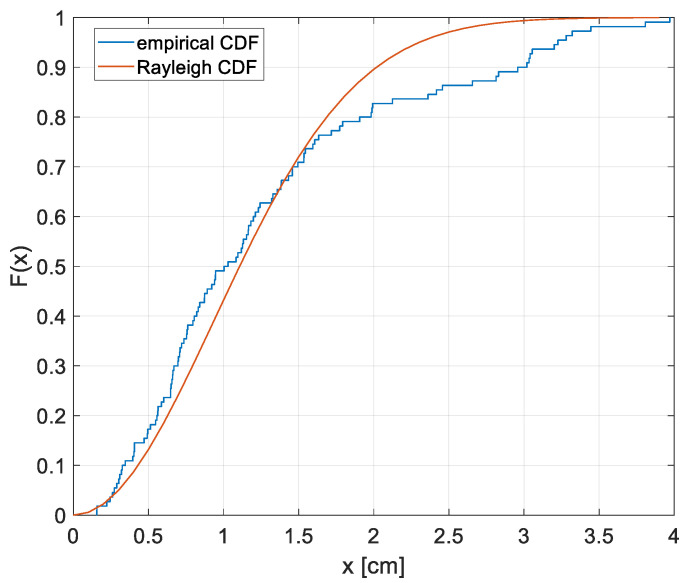
Empirical CDF F(x) of the positioning error (in blue) together with the CDF of a Rayleigh distribution; on the *x*-axis the positioning error in cm.

Figure 7b is a heatmap representing the error measured as the deviation between the ‘true positions’ and the ones derived with the positioning algorithm. In the map, the error values, in cm, are reported in each measurement point. Assuming a maximum admissible error equal to 5 cm, the proposed system well satisfies the requirement since the maximum obtained error is 3.97 cm while the mean error and the standard deviation of the error over the grid are 1.28 cm and 0.92 cm. The cumulative distribution function (CDF) F(x) of the positioning error is reported in Figure 8 as a blue line together with the CDF of a Rayleigh distribution; in the *x*-axis there is the error in cm.

The measurement area is the scaled version of a real one with scaling factor *sf* = 5 while the illumination in lx at the floor level is analogous to that which would occur in case of a greater distance between LEDs and receiver. Considering Equation (1), the received power $P_{\mathrm{Ri}}^{*}$ (the terms with apex ‘*’ refer to the wider tested area) would be equal to $P_{\mathrm{Ri}}$ (the terms without apex refer to the scaled tested area), since the higher distance between LEDs and receiver is compensated by a higher $P_{\mathrm{Ti}}^{*}$. Considering Equation (2) and all the terms which remain constant in the two situations, we can show that the measurement uncertainty of $d_{i}^{*}$, u($d_{i}^{*}$), satisfies the following u($d_{i}^{*}$) = $\sqrt{sf\;}u$($d_{i}$), (where u($d_{i}$) is the uncertainty of $d_{i}$). Considering the solution of the system in Equation (4), it is possible to show that, for the propagation of uncertainty, the measurement uncertainty of $x_{R}$ and $y_{R}$ will behave as u($x_{R}^{*}$) = $sf\sqrt{sf\;}u$($x_{R}$) (where u($x_{R}$) is the uncertainty of $x_{R})$, therefore the error will scale of a factor $sf\sqrt{sf}$ ≃ 11. This means that the average error of 1.28 cm in our set-up will be translated in an error of less than 15 cm in a wider deployment, which can be reputed a satisfactory result for indoor localization. The accuracy reached by IPSs is very variable, ranging from m or tens of cm in experimental tests (depending on the testing area dimensions) to mm in case of simulated tests. A preliminary investigation revealed that the achieved positioning accuracy, appropriately scaling the error in proportion to the dimensions of the measurement area, is comparable with that attained in other scientific works, envisaging also different localization approaches and technologies [5].

Moreover, since in the test bench, $f_{1}$, $f_{2}$ and $f_{3}$ are accurately generated, no substantial difference occurs between windowed and non-windowed estimations. Indeed, as explained in Section 5.2, the values of N, $f_{s}$, $f_{1}$, $f_{2}$ and $f_{3}$ are chosen to minimize spectral leakage in order to perform localization even without window, thus reducing the complexity of the algorithm. Finally, greatest errors are distributed in the highest part of the grid, this can be justified with a slight inclination of the floor of the measurement set-up in that area or with a non-perfect estimate of the parameter $K_{i}$ for ${\mathrm{LED}}_{1}$.

**Figure 9:**
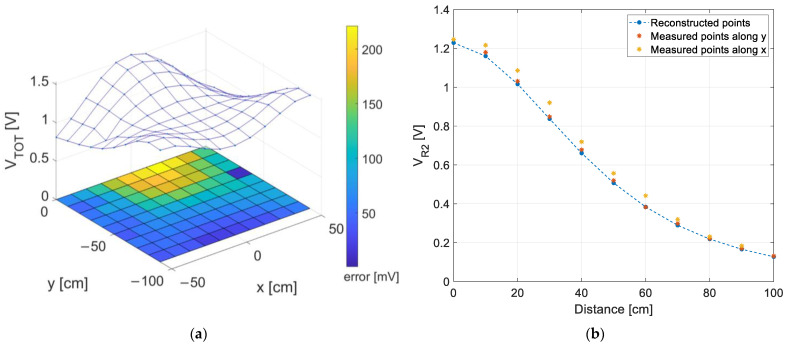
(**a**) The estimated RSSs distribution of the three LEDs as a 3D blue surface (i.e., the sum of the FFT-derived $V_{\mathrm{Ri}}$ in each measurement point, $V_{\mathrm{TOT}}$), on the (*x*,*y*) plane a heatmap reporting the absolute difference in mV between $V_{\mathrm{TOT}}$ and the RSSs recovered using (2) and the ‘true’ $d_{i}$. (**b**) Trend of the estimated $V_{R2}$ versus the distance: in blue the trend recovered using (2) and the ‘true’ $d_{i}$, the yellow and red marks are the FFT-extracted values moving respectively along *x*-axis and *y*-axis.

This aspect is further highlighted in Figure 9 where the total measured received optical power is shown. In particular, Figure 9a displays, as a 3D blue surface, the total RSSs distribution evaluated as the receiver voltage output,$\;V_{\mathrm{TOT}}$, accounting for the three LEDs contributions (i.e., the sum of the FFT-derived $V_{\mathrm{Ri}}$ in each measurement point). The heatmap on the (*x*,*y*) plane reports the absolute difference in mV between the extracted RSSs and the RSSs predicted by the model, i.e., derived by inverting (2) and by substituting to $d_{2}$ the known distance between solar cell and ${\mathrm{LED}}_{2}$, recovered from the ‘true’ coordinates ($x_{k}$,$y_{k}$) of the grid points. The color bar gives a qualitative information of the voltage error: the greatest error is observed in correspondence of ${\mathrm{LED}}_{1}$, confirming that the highest errors are probably due to the aforementioned reasons. Concerning ${\mathrm{LED}}_{2}$ and ${\mathrm{LED}}_{3}$, a good match between measured and predicted values can be found. For example, Figure 9b shows the trend of $V_{R2}$ with the distance from ${\mathrm{LED}}_{2}$: in blue the predicted trend recovered using (2) and the ‘true’ $d_{i}$, with yellow and red marks the FFT-extracted values moving respectively along *x*-axis and *y*-axis at steps of 10 cm.

These results confirm that the estimated $K_{i}$ well approximate the relationship between light intensity—and consequently measured voltage—and photogenerated current. Therefore, it can be assessed that the positioning inaccuracy noted in the tests is rather due to other sources of error as light reflections or non-perfect positioning of the receiver in the grid which led to a non-perfect estimation of $K_{1}$.

**Figure 10:**
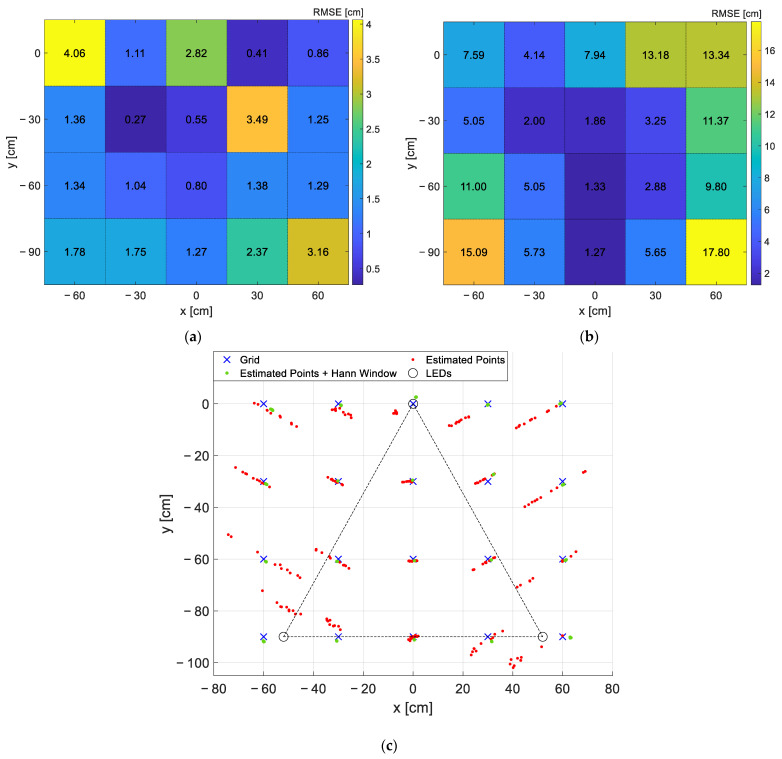
Results of the test performed with $f_{1}\;$= 1111 Hz instead of 1100 Hz in the 20 grid points equally spaced of 30 cm, collecting 10 measurements with Hann window and 10 without for each point. RMSEs in each grid position, respectively, with (**a**) and without (**b**) windowing; the values in the maps report the exact RMSEs in cm. (**c**) Qualitative grid representation: in blue the ‘true positions’ of the grid points, in green the coordinates estimated using the Hann window, in red the coordinates estimated without windowing operation.

To assess the capability of the proposed IP system to counteract possible clock inaccuracies, expected in a low-cost implementation, an additional test was performed introducing a very large misalignment between clocks, i.e., injecting an error of 1% in the frequency generation of ${\mathrm{LED}}_{1}$ (i.e., $f_{1}$ = 1111 Hz instead of 1100 Hz): the 2D positions are estimated using the proposed localization algorithm making a comparison between the RSSs estimated without and with Hann windowing. In this test the optical receiver is moved along the entire measurement area in steps of 30 cm (i.e., 12 30 cm × 30 cm squares, 20 measurement points), and collecting 10 measurements for each grid point. In Figure 10a,b, the RMSEs (root mean square error) for the two cases (with window in Figure 10a and without in Figure 10b) are reported. Figure 10c gives the same information on a grid representation, showing in red the points obtained without window and in green those obtained using the window. Moreover, in the same figure the ‘true’ positions of the grid points are represented in blue. It can be assessed that the usage of the window has the main effect of strongly reducing the variability of the results making the proposed system suitable even in case of large errors in the generation of the LED driving signals, as can be in case of real deployments where oscillators are used in place of extremely precise waveform generators. Indeed, the use of the Hann window and of the selected IP system setting ensures RMSE below the target value of 5 cm even in the presence of frequency deviations up to 1%.

Note that the dispersion of the results obtained without the Hann window, due to spectral leakage, is related to the random phase among the signals.

Finally, a further test was performed by shielding the measurement area in order to evaluate the effect that the external lights have on the localization accuracy (i.e., modulated artificial lights used for indoor lighting, background artificial lights). The material used is not anti-reflective since the goal of the shielding was only to hinder the external light sources and not to avoid reflections.

**Figure 11:**
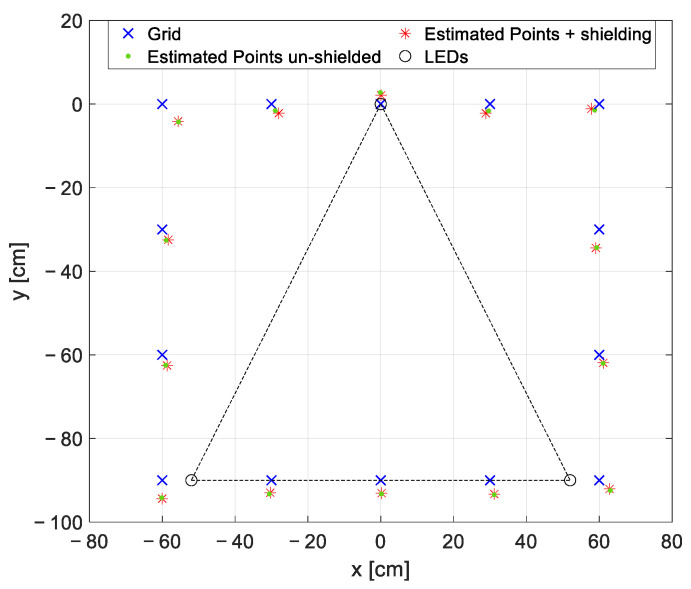
Qualitative grid representation of the estimated coordinates for the test performed shielding the measurement area and considering just the 14 perimeter points: in blue the ‘true positions’ of the grid points, in red and green, respectively, the coordinates estimated shielding and un-shielding the measurement area.

The optical receiver is moved with steps of 30 cm along the 14 points constituting the perimeter of the entire measurement area, since these points, for the arranged measurement set-up, are the most affected by the external light presence. The results are reported in Figure 11. As expected, the proposed VLP approach is reliable to additional modulated light sources, as the ones at 50 Hz employed for indoor lighting, and to the presence of background light. Indeed, the interference of external modulated lights is mitigated thanks to the accurate selection of the LEDs’ frequencies (i.e., not multiples of 50 Hz and much greater than 50 Hz so that the higher harmonics of the other lights sources are significantly attenuated and do not interfere with the spectrum of the light signal used for localization) and to the usage of the FFT, which selects only the RSSs at the specific LEDs’ frequencies. In this way, the alternated external light sources, together with the background light, contribute only as a constant value increasing the mean average illuminance under the LEDs, that can be discarded as a DC value in the FFT evaluation. This aspect becomes problematic only if it causes a significant reduction of the dynamic of the electronic front-end.

### 6.2. Node Self-Sufficiency Tests

The node is tested in the worst illumination condition, that is the center of the measurement grid in point (0,−60,0) where the minimum level of illuminance is experienced (i.e., 410 lx). The MCU is programmed to work in sleep mode, waking up periodically (i.e., every 15 min during the first test and every 10 min during the second test) to transmit via LoRa technology the estimated position of the target. The goal of the tests is to experimentally validate the energy self-sufficiency of the proposed architecture using the solar cell both as energy harvester and optical receiver for VLP, to this aim the voltage trend of the rechargeable Li-Ion battery, $V_{\mathrm{Li}-\mathrm{Ion}}$, is measured for the entire duration of the tests.

**Figure 12:**
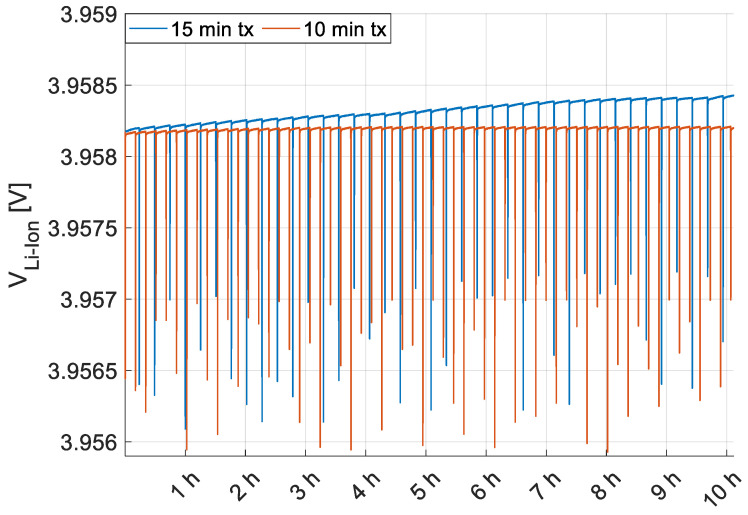
Li-Ion battery voltage behavior, $V_{\mathrm{Li}-\mathrm{Ion}}$, during two tests lasting 10 h each and performed in the grid point (0,−60,0) at the minimum level of illuminance (i.e., 410 lx). The blue plot corresponds to the case of radio transmissions every 15 min while the red plot refers to transmissions every 10 min.

The battery voltage behavior, monitored for approximately 10 h for each measurement campaign, is shown in Figure 12: the blue plot corresponds to the case of radio transmissions every 15 min, while the red plot refers to transmissions every 10 min, and the negative spikes are the voltage drops caused by the peak consumptions due to LoRa transmissions (i.e., 40 during the first test and 60 during the second test).

The increase of the battery level, net of node consumption, is minimal since the power delivered by the cell in the minimum lighting condition is around 305 µW; however, the difference between the voltage level at the end and at the beginning of the tests is positive in both cases. In particular, while with transmissions every 15 min, a battery charge, albeit small, is detectable, in the case of transmissions, every 10 min, a perfect balance between harvested and absorbed energy is achieved and an increasing trend can be detected only in the long period.

Thus, these results validate the energy self-sufficiency of the system even in case of minimum illuminance conditions and quite frequent transmissions. Indeed, one transmission every 10 min or at least 15 min can be considered a good transmission rate for those deployments where real-time localization is not required, as car tracking in a parking or goods inventory.

## 7. Conclusions

This paper presented a solar cell-based low power system featuring 2D VLP and autonomously powered by the indoor artificial lights exploited for the localization task.

Three modulated LED sources, univocally identified by a unique operating frequency, were used as anchors to infer the optical receiver position. In particular, the localization was performed by means of a low-complexity algorithm foreseeing FFT extraction of the received light intensities, optical channel estimation and lateration using just one measurement point (i.e., the center of gravity of the localization area subtended by the three LEDs) for channel parameter extraction. The 2D coordinates were derived directly on board exploiting a low-power microcontroller, then sent to a server using LoRaWAN as data transmission protocol. The system was equipped with an ultralow-power energy harvester and battery charger providing battery overvoltage and undervoltage protection, energy provisioning and a regulated power supply at 3.3 V for the node. A small-sized amorphous silicon solar cell, well-matching the spectral distribution of the used LEDs, was employed alternately as energy harvester and as optical receiver, in order to perform fully energy autonomous indoor localization.

Field tests were performed moving the receiver in fixed-length steps along an ad-hoc measurement set-up covering a localization area with dimensions 90 cm × 120 cm, divided into 10 cm × 10 cm squares identifying 130 distinct 2D positions in the plane. In order to make the proposed system less prone to inaccuracy in the generation of the driving signals, a Hann window was added before FFT and new tests were performed considering a frequency mismatch of 11 Hz in the generation of the signal driving ${\mathrm{LED}}_{1}$ (i.e., 1% frequency error). This test validates the effectiveness of the proposed IP methodology in case of worse clock accuracy as the one achievable in realistic deployments where the lighting system are likely driven by low-cost and low-complexity relaxation oscillators instead of more precise and more expensive signal generators, which would make the system not realizable in large scale. Moreover, the effect of external light sources on the proposed localization algorithm was evaluated. In all the tests, accurate positioning results with error lower than 5 cm were observed which can be considered compliant with the requirement of distinguishing between two adjacent points spaced by 10 cm.

Two additional tests were performed to assess the energy self-sufficiency of the node in the (0,−60,0) point, which is the one at lower illuminance (i.e., 410 lx). The Li-Ion battery voltage was monitored for 10 h with the three LEDs on and considering transmissions every 15 min and 10 min, which are compliant with quasi-real time monitoring tasks. The obtained results prove the fully energy autonomy of the target in the condition of worst illumination (i.e., harvested power in the order of hundreds of µW), satisfying both the harvesting and the localization tasks, thus gaining a node lifetime virtually infinite. However, since the tests demonstrate that the power delivered by the PV module is sufficient for supplying the node operations, the proposed system may be further engineered to be fully battery-less, embedding, at most, a small supercapacitor to manage the peak current absorptions of the radio transmissions. In this case, the fact of foreseeing an energy provisioning procedure becomes a strength with respect to similar nodes relying on a rechargeable/disposable battery as on-board energy storage.

In conclusion, the outcomes of these preliminary measurement campaigns suggest the adaptability of the system to IoT, Industry 4.0 and Smart Cities scenarios, where artificial lights are often available energy sources.

To further strengthen these results, field tests may be carried out in future collecting measurements in a wider indoor deployment and facing new challenges aimed at handling the mentioned weakness of the system, such as the parallelism between LEDs and receiver axes, the presence of obstacles and shadows, which are well-known limitations in VLP applications. Although the proposed methodology can manage inclinations in the order of ±10°, PV modules with better relative sensitivity can be selected and a hybrid positioning strategy can be implemented mounting an IMU on board in order to account for the inclination angles. Similarly, novel solutions different from the one proposed can be investigated to detect the presence of obstacles, completely or partially shading the light sources, and consequently to discard the result of the localization when not reliable, considering that in most cases these are transient disturbances or fix obstacles that can be accounted in a preliminary calibration phase.

However, despite these limitations, the system in the present form can find application in those scenarios where indoor 2D localization is required and the presence of obstacles is limited, such as localization of cars in a covered parking area, containers in a warehouse or people moving indoor.

## Data Availability

Not applicable.

## References

[1] Do T.H., Yoo M. (2016). An in-depth survey of visible light communication based positioning systems. Sensors.

[2] Zhuang Y., Hua L., Qi L., Yang J., Cao P., Cao Y., Yongpeng W., Thompson J., Haas H. (2018). A survey of positioning systems using visible LED lights. IEEE Commun. Surv. Tutor..

[3] Sadowski S., Spachos P. (2018). Rssi-based indoor localization with the internet of things. IEEE Access.

[4] Zafari F., Gkelias A., Leung K.K. (2019). A survey of indoor localization systems and technologies. IEEE Commun. Surv. Tutor..

[5] Gu F., Hu X., Ramezani M., Acharya D., Khoshelham K., Valaee S., Shang J. (2019). Indoor localization improved by spatial context—A survey. ACM Comput. Surv. (CSUR).

[6] Guo X., Ansari N., Hu F., Shao Y., Elikplim N.R., Li L. (2019). A survey on fusion-based indoor positioning. IEEE Commun. Surv. Tutor..

[7] Rahman A.B.M., Li T., Wang Y. (2020). Recent advances in indoor localization via visible lights: A survey. Sensors.

[8] Obeidat H., Shuaieb W., Obeidat O., Abd-Alhameed R. (2021). A review of indoor localization techniques and wireless technologies. Wirel. Pers. Commun..

[9] Mao W., Xie H., Tan Z., Liu Z., Liu M. (2020). High precision indoor positioning method based on visible light communication using improved Camshift tracking algorithm. Opt. Commun..

[10] Wei B., Xu W., Luo C., Zoppi G., Ma D., Wang S. SolarSLAM: Battery-free loop closure for indoor localisation. Proceedings of the 2020 IEEE/RSJ International Conference on Intelligent Robots and Systems (IROS).

[11] Andò B., Baglio S., Crispino R., Marletta V. (2021). An Introduction to Indoor Localization Techniques. Case of Study: A Multi-Trilateration-Based Localization System with User–Environment Interaction Feature. Appl. Sci..

[12] Jushi A., Pegatoquet A., Le T.N. Wind energy harvesting for autonomous wireless sensor networks. Proceedings of the 2016 Euromicro Conference on Digital System Design (DSD).

[13] Chamanian S., Uluşan H., Zorlu Ö., Baghaee S., Uysal-Biyikoglu E., Külah H. (2016). Wearable battery-less wireless sensor network with electromagnetic energy harvesting system. Sens. Actuators A Phys..

[14] Muncuk U., Alemdar K., Sarode J.D., Chowdhury K.R. (2018). Multiband ambient RF energy harvesting circuit design for enabling batteryless sensors and IoT. IEEE Internet Things J..

[15] Osorio de la Rosa E., Vázquez Castillo J., Carmona Campos M., Barbosa Pool G.R., Becerra Nuñez G., Castillo Atoche A., Ortegón Aguilar J. (2019). Plant microbial fuel cells–based energy harvester system for self-powered IoT applications. Sensors.

[16] Cappelli I., Parrino S., Pozzebon A., Salta A. (2021). Providing Energy Self-Sufficiency to LoRaWAN Nodes by Means of Thermoelectric Generators (TEGs)-Based Energy Harvesting. Energies.

[17] Rubes O., Chalupa J., Ksica F., Hadas Z. (2021). Development and experimental validation of self-powered wireless vibration sensor node using vibration energy harvester. Mech. Syst. Signal Process..

[18] Sharma H., Haque A., Jaffery Z.A. (2018). Solar energy harvesting wireless sensor network nodes: A survey. J. Renew. Sustain. Energy.

[19] Yue X., Kauer M., Bellanger M., Beard O., Brownlow M., Gibson D., Clark C., MacGregor C., Song S. (2017). Development of an indoor photovoltaic energy harvesting module for autonomous sensors in building air quality applications. IEEE Internet Things J..

[20] Pecunia V., Occhipinti L.G., Hoye R.L. (2021). Emerging Indoor Photovoltaic Technologies for Sustainable Internet of Things. Adv. Energy Mater..

[21] Bruzzi M., Cappelli I., Fort A., Pozzebon A., Vignoli V. (2022). Development of a Self-Sufficient LoRaWAN Sensor Node with Flexible and Glass Dye-Sensitized Solar Cell Modules Harvesting Energy from Diffuse Low-Intensity Solar Radiation. Energies.

[22] Cappelli I., Fort A., Pozzebon A., Tani M., Trivellin N., Vignoli V., Bruzzi M. (2022). Autonomous IoT Monitoring Matching Spectral Artificial Light Manipulation for Horticulture. Sensors.

[23] Wang Z., Tsonev D., Videv S., Haas H. Towards self-powered solar panel receiver for optical wireless communication. Proceedings of the 2014 IEEE International Conference on Communications (ICC).

[24] Wang Z., Tsonev D., Videv S., Haas H. (2015). On the design of a solar-panel receiver for optical wireless communications with simultaneous energy harvesting. IEEE J. Sel. Areas Commun..

[25] Liu Y., Chen H.Y., Liang K., Hsu C.W., Chow C.W., Yeh C.H. (2015). Visible light communication using receivers of camera image sensor and solar cell. IEEE Photonics J..

[26] Fan X., Leon-Salas W.D. A circuit for simultaneous optical data reception and energy harvesting. Proceedings of the 2017 IEEE 60th International Midwest Symposium on Circuits and Systems (MWSCAS).

[27] Hsu C.W., Wu J.T., Wang H.Y., Chow C.W., Lee C.H., Chu M.T., Yeh C.H. (2016). Visible light positioning and lighting based on identity positioning and RF carrier allocation technique using a solar cell receiver. IEEE Photonics J..

[28] Chen X., Min C., Guo J. (2017). Visible light communication system using silicon photocell for energy gathering and data receiving. Int. J. Opt..

[29] Chaabna A., Babouri A., Zhang X. An indoor positioning system based on visible light communication using a solar cell as receiver. Proceedings of the International Conference in Artificial Intelligence in Renewable Energetic Systems.

[30] Umetsu Y., Nakamura Y., Arakawa Y., Fujimoto M., Suwa H. Ehaas: Energy harvesters as a sensor for place recognition on wearables. Proceedings of the 2019 IEEE International Conference on Pervasive Computing and Communications (PerCom).

[31] Rizk H., Ma D., Hassan M., Youssef M. Indoor Localization using Solar Cells. Proceedings of the 2022 IEEE International Conference on Pervasive Computing and Communications Workshops and other Affiliated Events (PerCom Workshops).

[32] Chen H., Guan W., Li S., Wu Y. (2018). Indoor high precision three-dimensional positioning system based on visible light communication using modified genetic algorithm. Opt. Commun..

[33] Zhang H., Cui J., Feng L., Yang A., Lv H., Lin B., Huang H. (2019). High-precision indoor visible light positioning using modified momentum back propagation neural network with sparse training point. Sensors.

